# Pediatric T-cell lymphoblastic lymphoma with t(9;17)(q34;q23)-associated N-terminally truncated NOTCH1 activation: a case report

**DOI:** 10.1007/s12185-026-04263-6

**Published:** 2026-09-02

**Authors:** Ayako Yamamori, Daichi Sajiki, Yusuke Tsumura, Asahito Hama, Manabu Wakamatsu, Nao Yoshida, Daiki Yamashita, Kotaro Narita, Shinsuke Kataoka, Yusuke Okuno, Hideki Muramatsu, Yoshiyuki Takahashi

**Affiliations:** 1 Department of Pediatrics, Nagoya University Graduate School of Medicine, Nagoya, Aichi Japan; 2 Department of Hematology and Oncology, Children’s Medical Center, Japanese Red Cross Aichi Medical Center Nagoya First Hospital, Nagoya, Aichi Japan; 3 Department of Virology, Nagoya City University Graduate School of Medical Sciences, Nagoya, Aichi Japan

**Keywords:** T-cell lymphoblastic lymphoma, Truncated NOTCH1, 9q34, *MIR142HG::NOTCH1*

## Abstract

T-cell lymphoblastic lymphoma (T-LBL) is an aggressive malignancy that comprises 20% of pediatric non-Hodgkin lymphoma cases. Most T-LBLs harbor *NOTCH1* point mutations or small indels, which are generally associated with favorable outcomes. In contrast, the clinical impact of structural rearrangements involving *NOTCH1* remains poorly understood. We describe the case of a 6-year-old boy with T-LBL carrying a *MIR142HG::NOTCH1* fusion from a t(9;17)(q34;q23) translocation, resulting in a truncated, constitutively active NOTCH1 protein. The patient experienced an aggressive course and died shortly after cord blood transplantation. Literature review suggests that 9q34 translocations in T-LBL portend poor prognosis, highlighting truncated *NOTCH1* as a potential therapeutic target.

**Supplementary Information:**

The online version contains supplementary material available at https://doi.org/10.1007/s12185-026-04263-6.

## Introduction

T-cell lymphoblastic lymphoma (T-LBL) is an aggressive malignancy of immature T cells, comprising 20% of pediatric non-Hodgkin lymphoma cases [1]. Genetic alterations in *NOTCH1*, including point mutations and small indels, are frequently observed in patients with T-LBL/T-cell acute lymphoblastic leukemia (T-ALL) and are typically associated with favorable prognoses [2]. However, structural chromosomal abnormalities are also common in T-LBL [1]. Patients with T-LBL harboring t(9;17)(q34;q23) translocations have been sporadically reported, but detailed genetic characterization has been lacking [3–6]. Herein, we report a child with T-LBL harboring *MIR142HG::NOTCH1* fusion gene, resulting from t(9;17)(q34;q23), who relapsed and ultimately succumbed to the disease. Additionally, we conducted a systematic literature review on T-LBL patients with t(9;17)(q34;q23), which demonstrated an extremely poor prognosis.

### Case

A 6-year-old boy presented with dyspnea and recurrent coughing. The detailed clinical course of this patient was previously reported in a Japanese-language case report [7]. Contrast-enhanced computed tomography (CT) scan showed left pleural and pericardial effusions and an anterior mediastinal mass (12 cm) compressing the left bronchus. ^18^F-fluorodeoxyglucose positron emission tomography (FDG-PET) revealed an abnormal FDG accumulation in the mediastinal tumors, pleura, pericardium, and lymph nodes in the left supraclavicular, left axillary, and para-aortic areas. The pathological cytology of the pleural fluid showed proliferation of atypical cells with a high nuclear content-to-cytoplasmic volume ratio. Flow cytometry revealed positivity for CD2, CD3, CD4, and CD8 and negativity for CD79a and TdT. Chromosomal analysis showed a normal karyotype. Based on these findings, he was diagnosed with stage III T-LBL.

After induction chemotherapy, FDG-PET confirmed metabolic complete remission. However, during subsequent chemotherapy, FDG accumulation reappeared in the anterior mediastinum. Thoracoscopic biopsy confirmed relapse, with chromosomal analysis revealing the presence of 46,XY,t(9;17)(q34;q23). He was diagnosed with T-LBL recurrence. Local irradiation (30.6 Gy in 17 fractions) temporarily reduced tumor size, but blasts appeared in peripheral blood (PB). He did not respond to subsequent chemotherapy and underwent cord blood transplantation from a human leukocyte antigen-matched unrelated donor with a conditioning regimen of melphalan (180 mg/m^2^) and total-body irradiation (12 Gy), following short-term methotrexate and tacrolimus as a graft-versus-host disease prophylaxis. Although engraftment was achieved, blasts (12%) reappeared in PB by day + 21. A FDG-PET scan on day + 26 confirmed disease progression, with FDG accumulation in the cervical lymph nodes, left pleura, and systemic bone. He eventually died of the disease on day + 70 after transplantation.

**Fig. 1:**
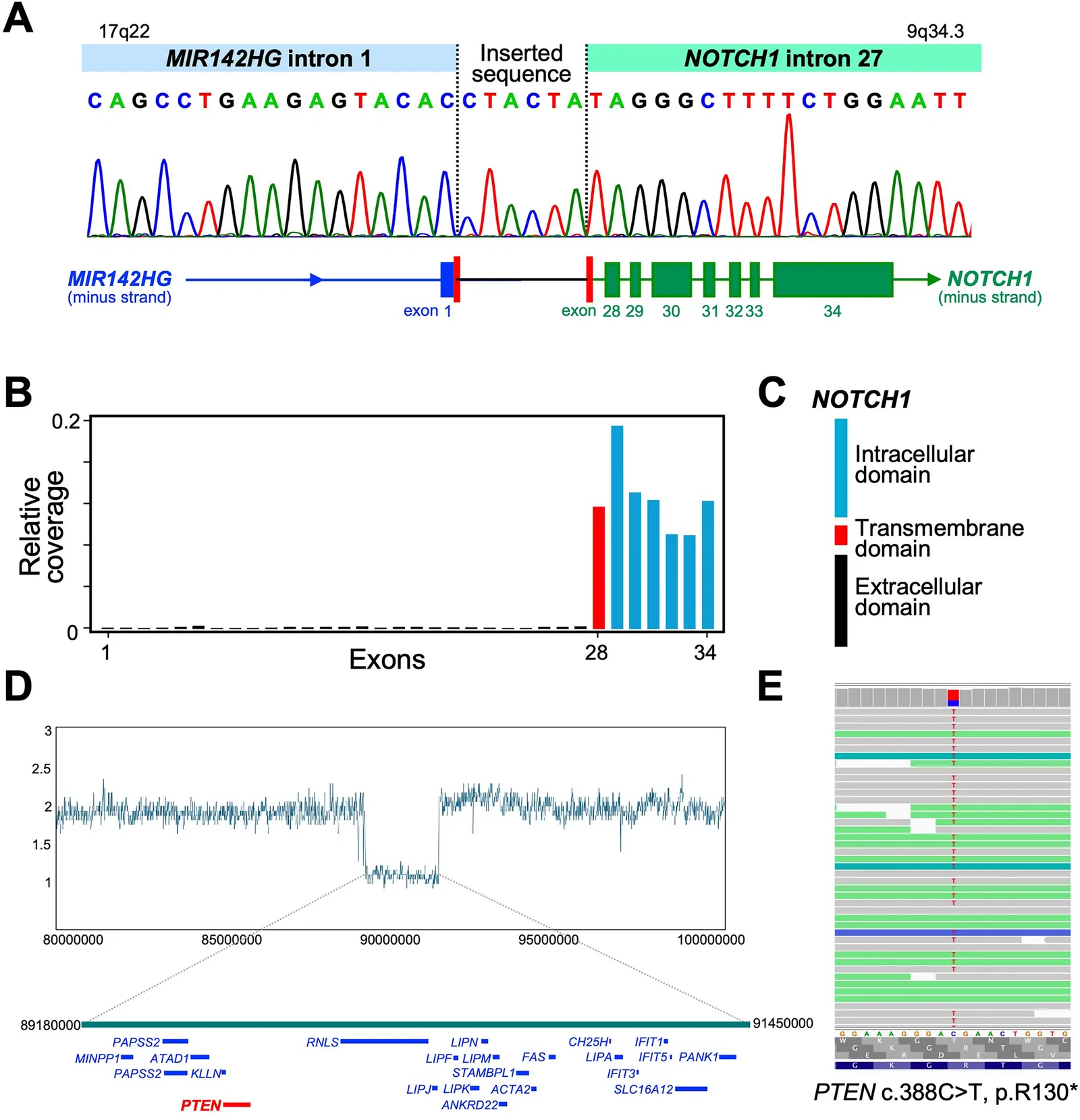
Comprehensive genetic analysis revealing *NOTCH1* translocation and *PTEN* alterations. **A** Confirmation of the translocation via Sanger sequencing, which showed breakpoints at intron 1 of *MIR142HG* and intron 27 of *NOTCH1*. **B** RNA sequence coverage of *NOTCH1* in the patient, relative to *GAPDH*. The transmembrane domain (red, exon 28) and the intracellular domain (blue, exons 29–34) had a higher coverage than the extracellular domain (black, exons 1–27). The fusion transcript joins the non-coding *MIR142HG* exon 1 (5′) to *NOTCH1* exon 28 (3′); translation is predicted to initiate at an internal methionine within *NOTCH1* exon 28 (Met1727), in the native *NOTCH1* reading frame, yielding an N-terminally truncated NOTCH1 protein composed of the transmembrane and intracellular domains. **C** Illustration of the *NOTCH1* structure, which comprised intracellular domain (blue line), transmembrane domain (red line), and extracellular domain (black line). **D** Copy number analysis of chromosome 10 via whole-genome sequencing revealed a 2.3-Mb hemizygous deletion at 10q23.3, which includes the whole *PTEN* region. **E** *PTEN* hemizygous stop-gain variant (c.388C > *T*, p.R130*, variant allele frequency of 0.83) identified via whole-genome sequencing and displayed using Integrative Genomics Viewer

RNA sequencing and whole-genome sequencing (WGS) were performed on the relapse tumor samples (Supplemental Methods). RNA sequencing and WGS confirmed the presence of t(9;17)(q34;q23) translocation, which resulted in a fusion between intron 27 of *NOTCH1* on chromosome 9 and intron 1 of *MIR142HG* on chromosome 17 (*MIR142HG::NOTCH1*) (Fig. 1A). RNA sequencing further revealed elevated mRNA expression in exons 28–34 of *NOTCH1*, which encode the transmembrane and intracellular domains, suggesting the production of a truncated NOTCH1 protein lacking the extracellular domain (Fig. 1B, C). In addition, WGS identified a 2.3-Mb hemizygous deletion on chromosome 10q23.31–q23.33 encompassing the *PTEN* region (chr10:89,180,000–91,450,000, GRCh37/hg19; Fig. 1D), together with a nonsense *PTEN* variant on the retained allele (NM_000314, c.388C > *T*, p.R130*, variant allele frequency: 0.83; Fig. 1E), consistent with biallelic *PTEN* inactivation. Furthermore, hemizygous deletions in *CDKN2A/B* and *IKZF2* were detected (Supplemental Fig. 1 A, B).

A systematic literature review identified four articles describing eight patients with T-LBL/T-ALL harboring t(9;17)(q34;q23) [3–6] (Supplemental Methods, Supplemental Fig. 2, Table 1). Including our case, the cohort comprised nine patients (five boys and four girls) with a median age of 10 years (range: 5–15 years), eight of whom developed stage III T-LBL and the remaining patient had T-ALL. Clinical data were not available for one patient, for whom only cytogenetic findings had been reported, whereas seven of the remaining eight patients died of the primary disease.

**Table 1:** Clinical course of children with T-LBL/T-ALL harboring t(9;17)(q34;q23) abnormalities

No	Age,	Sex	Disease	Karyotype	Outcome	References
	Years					
1	14	F	T-LBL	46,XX,t(9;17)(q34;q23)	DOD	3
2	15	M	T-LBL	46,XY,−9,del(6)(q13q21),t(9;17)(q34;q23),+der(9)t(9;17)(q34;q23)	DOD	3
3	10	M	T-LBL	47,XY,+19,t(9;17)(q34;q23)	DOD	3
4	14	F	T-LBL	46,XX,t(9;17)(q34;q23)	DOD	4
5	7	M	T-LBL	49,XY,-1,+der(1)t(1;?)(p36;?),t(9;17)(q34;q23),+14,+mar1,+mar2	DOD	4
6	5	F	T-LBL	47,XX,t(9;17)(q34;q23),+der(17)t(9;17)(q34;q23)	CR	4
7	8	M	T-LBL	47,XY,t(9;17)(q3?4;q2?3),+20	DOD	5
8	13	F	T-ALL	46,XX,t(9;17)(q34;q23)	NA	6
**9**	**6**	**M**	**T-LBL**	**46,XY,t(9;17)(q34;q23)**	**DOD**	**Current case**

Bold indicates the present case

M, male; F, female; T-LBL, T-cell lymphoblastic lymphoma; T-ALL, T-cell acute lymphoblastic leukemia; DOD, died of the disease; CR, complete remission; NA, not available

## Discussion

*MIR142HG::NOTCH1* has recently been reported by Kroeze et al*.* [8] as a recurrent fusion in two pediatric T-LBL cases (TLBL042 and TLBL058), identified through RNA sequencing and confirmed by whole-genome sequencing, although no karyotypic information was available in that cohort. Clinically, our patient shared the characteristic features of the cases reported by Kroeze et al., including a bulky mediastinal mass with pleural and pericardial effusion and the absence of bone marrow, peripheral blood, and central nervous system involvement. Our case extends these observations by (i) providing the first karyotypic correlate, t(9;17)(q34;q23), for the *MIR142HG::NOTCH1* fusion; (ii) demonstrating concurrent *PTEN* hemizygous deletion combined with an R130* nonsense mutation; and (iii) placing this fusion within a systematic review of 9q34-translocated T-LBL cases, which collectively indicate an extremely poor prognosis.

Our case highlights the potential oncogenic role of *MIR142HG::NOTCH1* fusion gene in T-LBL harboring t(9;17)(q34;q23). The eight patients with 9q34 translocations identified in the literature review likely exhibited constitutive activation of the NOTCH1 signaling pathway. Although *NOTCH1* gene alterations are generally associated with a favorable prognosis in T-LBL [1], none of these patients achieved long-term survival, suggesting that 9q34 translocations represent a poor prognostic factor in T-LBL.

*NOTCH1* was initially identified in T-ALL with t(7;9) translocations [9]. NOTCH1 receptor signaling is mediated by ligand–receptor interactions that lead to conformational changes in the LIN-12/NOTCH1 repeat domain and dissociation of the heterodimerization (HD) domains, exposing a normally protected metalloproteinase cleavage site at the C-terminus of the HD domain. This mechanism leads to S2 cleavage of the NOTCH1 protein by metalloproteinases, and the cleaved protein is further processed (S3 cleavage) by the γ-secretase complex. The cytoplasmic domain of NOTCH1, released from the transmembrane region by S3 cleavage, is translocated to the nucleus where it forms a transcriptional complex with the DNA binding protein RBPJ/CSL to activate the expression of specific genes [10].

NOTCH1 functions as a master regulator of T-cell lineage commitment during thymic development, and this lineage specificity is reflected in the distribution of *NOTCH1* alterations across lymphoid malignancies. Activating *NOTCH1* mutations, typically clustering in the heterodimerization (HD) or PEST domains, are detected in approximately 55–60% of pediatric T-LBL cases [1], whereas they are rarely observed in B-cell precursor lymphoblastic lymphoma (B-LBL). In pediatric T-LBL, *NOTCH1* point mutations and small indels have generally been associated with a favorable prognosis [1, 2]. In contrast, the clinical significance of structural *NOTCH1* rearrangements—including t(7;9)(q34;q34.3), t(9;14)(q34.3;q11.2), and t(9;17)(q34;q23)—remains uncertain because of their rarity, and emerging data from our case and previously reported 9q34-translocated T-LBL cases suggest that such rearrangements may be associated with more aggressive clinical behavior.

In our case, the *MIR142HG::NOTCH1* fusion transcript retains *NOTCH1* exons 28–34, encoding the transmembrane and intracellular domains, while the upstream sequences encoding the extracellular domain—including the LIN-12/NOTCH1 repeats and heterodimerization domain that together constitute the negative regulatory region (NRR)—are lost. Translation is predicted to initiate at an internal methionine within exon 28 (Met1727), yielding an in-frame truncated NOTCH1 receptor. This configuration closely resembles the ΔE-type *NOTCH1* variants previously described in T-cell neoplasms, in which loss of the NRR permits ligand-independent S2 cleavage by ADAM metalloproteases, followed by γ-secretase-mediated S3 cleavage, releasing the NOTCH1 intracellular domain (NICD). The same activation mechanism has been documented in T-ALL with the t(7;9)(q34;q34.3) translocation [2] and in intragenic *NOTCH1* deletions [1], and we infer it to operate in our case as well. Because the rearrangement places *NOTCH1* immediately downstream of *MIR142HG*, the fusion is most likely driven by promoter hijacking by the *MIR142HG* locus. RNA-seq read coverage supports this model: the fusion transcript is robustly expressed, whereas signal from the wild-type *NOTCH1* allele is minimal (Fig. 1B). H3K27ac enrichment at the *MIR142HG* locus (Supplemental Fig. 3) suggests that additional *cis*-regulatory elements within this region may further sustain transcription of the fusion. Notably, *MIR142HG* hosts miR-142, one of the most abundant microRNAs in hematopoietic cells, and is highly transcribed in T-lineage cells [11], consistent with the proposed promoter hijacking mechanism in this disease context.

In our patient, *PTEN* was affected by two concurrent lesions: a 2.3-Mb hemizygous deletion at 10q23.31–q23.33 encompassing the entire *PTEN* locus, together with a nonsense mutation (p.R130*, variant allele frequency 0.83) on the retained allele, indicating biallelic *PTEN* inactivation and predicting constitutive activation of the PI3K/AKT signaling pathway. *PTEN* loss has been reported in approximately 8–17% of pediatric T-cell lymphoblastic neoplasms and often co-occurs with *NOTCH1* alterations [1, 12]. Mechanistically, NOTCH1 normally represses *PTEN* transcription through HES1, and loss of *PTEN* generates a NOTCH1-independent proliferation signal via the PI3K/AKT pathway; accordingly, T-cell lymphoblasts with biallelic *PTEN* inactivation can sustain cell growth even when NOTCH1 signaling is suppressed [12]. The coexistence of biallelic *PTEN* inactivation with constitutive NOTCH1 activation therefore provides a plausible biological explanation for the aggressive clinical course and refractoriness to conventional therapy in our patient and may partly reconcile the apparent discrepancy between the presence of a *NOTCH1*-activating lesion and the unfavorable outcome.

This study had several limitations. First, in vitro functional experiments were not conducted. However, previous studies have shown fusion gene mechanisms resulting in the production of a constitutively active NOTCH1 protein lacking the N-terminal region [13]. This indicates a mechanism similar to that in our case. Second, our literature review identified T-LBL cases with translocations involving the 9q34 region, but the presence of the *NOTCH1* fusion gene in these cases has not been confirmed. Further research is needed to validate these findings and to explore broader implications of 9q34 translocations in T-LBL.

In conclusion, our case revealed the potential oncogenic role of *MIR142HG::NOTCH1* fusion gene in T-LBL with t(9;17)(q34;q23) abnormalities. However, more studies are necessary to confirm these findings and to elucidate the significance of 9q34 translocations in T-LBL.

## Supplementary Information

Below is the link to the electronic supplementary material.

Supplementary file1 (DOCX 1278 kb)

## Data Availability

Sequencing data have been deposited at the Japanese Genotype–phenotype Archive, operated by the DNA Data Bank of Japan Center, under accession number JGAS000729.
