# Supplementary material for: Pediatric T-cell lymphoblastic lymphoma with t(9;17)(q34;q23)-associated N-terminally truncated NOTCH1 activation: a case report
Source: Int J Hematol. 2026 Sep 2;124(5):747–51. doi: 10.1007/s12185-026-04263-6 (PMC13719881; doi:10.1007/s12185-026-04263-6)
Supplement: Supplementary file 1 [file 12185_2026_4263_MOESM1_ESM.docx]

Supplementary Information

[Supplemental Methods	2](#_Toc216824764)

[Sample	2](#_Toc216824765)

[RNA sequencing	2](#_Toc216824766)

[Whole-genome sequencing	2](#_Toc216824767)

[Systematic literature review	3](#_Toc216824768)

[Supplemental Figures	4](#_Toc216824769)

[Supplemental Figure 1. Copy number abnormalities of CDKN2A/B and IKZF2 via WGS analysis.	4](#_Toc216824770)

[Supplemental Figure 2. Scheme of the literature review about patients with T-LBL harboring t(9;17)(q34;q23) abnormalities	5](#_Toc216824771)

[Supplemental Figure 3. UCSC Genome Browser view of the MIR142HG genomic region	6](#_Toc216824772)

[Supplemental figure legends	7](#_Toc216824773)

[Supplemental References	8](#_Toc216824774)

# Supplemental Methods

## Sample

Fresh-frozen tissues obtained via mediastinal lesion biopsy were used for DNA and RNA extraction. Genomic DNA was extracted using the QIAamp DNA Blood Mini Kit (QIAGEN, Hilden, Germany), according to the manufacturer’s instructions. Total RNA was extracted from the tumor samples of patients with bone marrow relapse using the RNeasy Mini Kit (QIAGEN, Hilden, Germany), according to the manufacturer’s instructions. The RNA integrity score (RNA integrity number equivalent; RINe) was assessed using RNA ScreenTape with the TapeStation 2200 system (Agilent, Santa Clara, CA, the USA).

## RNA sequencing

RNA sequencing was performed according to our previous report.^1^ Briefly, non-directional sequencing libraries were prepared and run on the Illumina next-generation sequencing platform (Illumina, San Diego, CA). The obtained reads were aligned with the hg19 human reference sequence using Tophat2 (version 2.0.14) and its fusion search option (TopHat-Fusion) for detecting fusion genes.^2,3^

## Whole-genome sequencing

Whole-genome sequencing was performed on the Illumina NovaSeq 6000 platform as paired-end 150-bp reads using libraries with an average insert size of 350 bp and prepared with the TruSeq DNA PCR-Free kit (Illumina, Inc., San Diego, CA, the USA). Sequencing data were aligned to the hg19 reference genome using bwa mem command (BWA, http://bio-bwa.sourceforge.net/) with -M option. Polymerase chain reaction duplicates were removed using Picard tools (http://broadinstitute.github.io/picard/). Single nucleotide variants and short insertion and deletion were detected and annotated using VarScan and ANNOVAR, respectively. Copy number analysis was performed using whole-genome sequencing with an in-house pipeline. The whole genome was segmented into 10-kb bins, and the median coverage of each bin was compared with the mean of the median coverage of all bins.

## Systematic literature review

We conducted a systematic literature review about case reports on patients with T-cell lymphoblastic lymphoma (T-LBL) / T-cell acute lymphoblastic leukemia (T-ALL) harboring t(9;17)(q34;q23) abnormalities using a combination of controlled vocabulary, if possible, and keywords as per the Preferred Reporting Items for Systematic Reviews and Meta-Analyses guidelines (<http://www.prisma-statement.org>). The PubMed database ([https://pubmed.ncbi.nlm.nih.gov/advanced/) and](https://pubmed.ncbi.nlm.nih.gov/advanced/)%20and) Google Scholar database (<https://scholar.google.co.jp/schhp?hl=ja>) were searched for articles published from the time of its inception until September 2025. The terminologies searched on the PubMed database used in the search were (T-lymphoblastic lymphoma OR T-LBL OR T-cell lymphoblastic leukemia/lymphoma) AND humans. The Boolean operator “AND” helped combine these phrases and narrow the search. The terminologies found on Google Scholar that were used in the search were “t(9;17)(q34;q23)”. This research identified 2577 electronic papers. Among them, 83 review articles, 12 articles about randomized controlled trials (excluded because they did not provide cytogenetic study results for individual cases), 11 textbook chapters, 5 articles not written in the English language, and 4 conference abstracts were excluded. Of the 2462 articles retrieved, all underwent abstract analysis. Among them, 2072 articles about other diseases were excluded, and 390 articles were selected for further detailed full text evaluation. Among them, 386 articles were excluded due to reasons such as focusing on pharmaceutical development and evaluation, different chromosomal abnormalities, or different diagnoses. Therefore, we selected four articles which describe eight patients with stage T-LBL/T-ALL harboring t(9;17)(q34;q23) abnormalities (**Supplemental Figure 2**).^4-7^

# Supplemental Figures

## Supplemental Figure 1. Copy number abnormalities of CDKN2A/B and IKZF2 via WGS analysis.

## Supplemental Figure 2. Scheme of the literature review about patients with T-LBL/T-ALL harboring t(9;17)(q34;q23) abnormalities

## Supplemental Figure 3. UCSC Genome Browser view of the MIR142HG genomic region

## Supplemental figure legends

**Supplemental Figure 1. Copy number abnormalities of *CDKN2A/B* and *IKZF2* via whole-genome sequencing (WGS) analysis.**

(a) The patient’s copy number analysis result around *CDKN2A/B* via WGS. The patient had the long-read deletion on chromosomes 21,890,000–22,259,999 (hg 19) containing the whole *CDKN2A/B.*
(b) The patient’s copy number analysis result around *IKZF2B* via WGS. The patient had deletions on chromosomes 213,885,511–213,977,235 and 213,990,645–214,111,432 (hg 19), including extensive deletions in two parts of *IKZF2.*

**Supplemental Figure 2. Scheme of the literature review about patients with T-LBL/T-ALL harboring t(9;17)(q34;q23) abnormalities**

The literature review was conducted using the PubMed database (<https://pubmed.ncbi.nlm.nih.gov/advanced/>) from the start of the study to September 30th, 2025, and the Google Scholar search engine (<https://scholar.google.co.jp/schhp?hl=ja>) (during the search in September 2025). Four literatures describing the clinical course of eight patients with T-LBL/T-ALL harboring t(9;17)(q34;q23) abnormalities were described.

**Supplemental Figure 3. UCSC Genome Browser view of the *MIR142HG* genomic region.**

UCSC Genome Browser (https://genome.ucsc.edu/) screenshot of the chr17q22-q23 locus (hg19, chr17:56,385,000–56,435,000), showing the *MIR142HG* transcript contained within the broader overlapping *TSPOAP1-AS1* lncRNA, together with adjacent genes (*TSPOAP1*, *SUPT4H1*, *RNF43*) and the Layered H3K27Ac track from seven ENCODE cell lines marking active regulatory elements across the region. The image was obtained on 14 May 2026. Data usage follows the guidelines of the UCSC Genome Browser and the ENCODE Consortium.

# Supplemental References

1.	Ichikawa D, Yamashita K, Okuno Y, Muramatsu H, Murakami N, Suzuki K, et al. Integrated diagnosis based on transcriptome analysis in suspected pediatric sarcomas. NPJ Genom Med. 2021;6(1):49.

2.	Kim D, Salzberg SL. TopHat-Fusion: an algorithm for discovery of novel fusion transcripts. Genome Biol. 2011;12(8):R72.

3.	Kim D, Pertea G, Trapnell C, Pimental H, Kelley R, Salzberg S. TopHat2: accurate alignment of transcriptomes in the presence of insertions, deletions and gene fusions. Genome Biol. 2013;14(4):R36.

4.	Kaneko Y, Frizzera G, Maseki N, Sakurai M, Komada Y, Sakurai M, et al. A novel translocation, t(9;17)(q34;q23), in aggressive childhood lymphoblastic lymphoma. Leukemia. 1988;2(11):745–8

5.	Shikano T, Ishikawa Y, Naito H, Kobayashi R, Nakadate H, Hatae Y, et al. Cytogenetic characteristics of childhood non-Hodgkin lymphoma. Cancer. 1992;70(3):714–9.

6.	Lones MA, Heerema NA, Le Beau MM, Sposto R, Perkins SL, Kadin ME, et al. Chromosome abnormalities in advanced stage lymphoblastic lymphoma of children and adolescents: a report from CCG-E08. Cancer Genet Cytogenet. 2007;172(1):1–11.

7.	Coccé MC, Alonso CN, Rossi JG, Bernasconi AR, Rampazzi MA, Felice MS, et al. Cytogenetic and Molecular Findings in Children with Acute Lymphoblastic Leukemia: Experience of a Single Institution in Argentina. Mol Syndromol. 2015;6(4):193–203.
